# Supplementary material for: Intracellular Signaling Responses Induced by Radiation within an In Vitro Bone Metastasis Model after Pre-Treatment with an Estrone Analogue
Source: Cells. 2021 Aug 17;10(8):2105. doi: 10.3390/cells10082105 (PMC8394480; doi:10.3390/cells10082105)
Supplement: Supplementary file 1 [file cells-10-02105-s001.zip › cells-1263444-supplementary.pdf]

Supplementary data

Helena et al 2021

Experimental dose-response curves determined the lowest ESE-16 concentration to induce significant cell death and the lowest radiation dose to significantly reduce cell viability to be used in combination in this study. Flow cytometric evaluation of cell cycle progression and Annexin V-FITC detection for apoptosis induction were performed. DU 145- and MDA-MB-231 cells were exposed to an ESE-16 concentration range (¼ GI50, ½ GI50, ¾ GI50 and GI50) for 48-h or a radiation dose range (4 Gy, 6 Gy and 8 Gy) followed by a 24-h incubation period.

Cell cycle progression in DU 145- and MDA-MB-231 cells exposed to ESE-16 or radiation is represented in Figure A1. In DU 145 cells, at ¼ GI50 (0.156 µM) a significant increase in sub-G1 cells (17.81 ± 2.46%) was observed when compared to DMSO (5.82 ± 1.21%). At ½ GI50 (0.313 µM), significant increases in the sub-G1- (42.94 ± 3.17%) and G2/M populations (34.47 ± 3.14%) were observed with a significant decrease in G1 cells (11.94 ± 2.04%). At 4 Gy, a significant increase in G2/M cells (39.81 ± 6.48%) and significant decreases in G1- (40.25 ± 0.78%) and S cells (9.84 ± 2.34%) were observed when compared to cells propagated in medium only. In MDA-MB-231 cells, no statistically significant differences were evident at ¼ GI50 (0.117 µM). At ½ GI50 (0.235 µM), significant increases in sub-G1- (29.88 ± 3.28%) and G2/M cells (34.43 ± 2.28%) were observed with a significant decrease in G1 cells (15.18 ± 6.49%) when compared to DMSO. At 4 Gy, a significant increase in G2/M cells (36.12 ± 4.98%) with a significant decrease in G1 cells (42.94 ± 2.81%) was observed when compared to cells propagated in medium only.

Annexin V-FITC apoptosis detection in DU 145- and MDA-MB-231 cells exposed to ESE-16 or radiation is represented in Figure A2. In DU 145 cells, no statistically significant differences were noted at ¼ GI50 (0.156 µM). At ½ GI50 (0.313 µM), a significant decrease in cell viability (63.62 ± 3.61%) with a concurrent significant increase in apoptosis (35.57 ± 3.76%) was observed when compared to DMSO. No statistically significant differences were noted when DU 145 cells were irradiated at 4 Gy. In MDA-MB-231 cells, no statistically significant differences were noted at ¼ GI50 (0.117 µM). At ½ GI50, ¾ GI50 and GI50 similar trends were observed in MDA-MB-231 cells as DU 145 cells with significant reductions in cell viability and significant increases in apoptosis when compared to DMSO. At ½ GI50 (0.235 µM), 78.05 ± 2.39% of cells were viable and 19.15 ± 0.99% of cells were apoptotic. At 4 Gy, a significant decrease in viable cells (74.18 ± 1.52%) and a significant increase in apoptotic cells (21.08 ± 2.35%) were observed when compared to cells propagated in medium only.

ESE-16 concentrations between ¼ GI50 and ½ GI50 were deemed to be the lowest concentrations to induce notable G2/M phase arrest and apoptosis in DU 145- and MDA-MB-231 cells (Pre-sensitization dose = (¼ GI50 + ½ GI50)/2). ESE-16 concentrations of 0.235 µM and 0.176 µM were therefore selected for further exposure in DU-145- and MDA-MB-231 cells respectively. A radiation dose of 4 Gy revealed to be the lowest dose to decrease cell viability to approximately 80% in both cell lines. Thus, DU 145 cells, RAW 264.7 macrophages and HUVECs were exposed to 0.235 µM ESE-16 and MDA-MB-231 cells were exposed to 0.176 µM ESE-16 for 24-h prior to a single dose of 4 Gy radiation (Table A1).

Table S1. ESE-16 concentrations and radiation doses used in all further combination studies.

Cell lines	ESE-16 (µM)	Radiation (Gy)
DU 145, RAW 264.7, HUVEC	0.235	4
MDA-MB-231	0.176	4

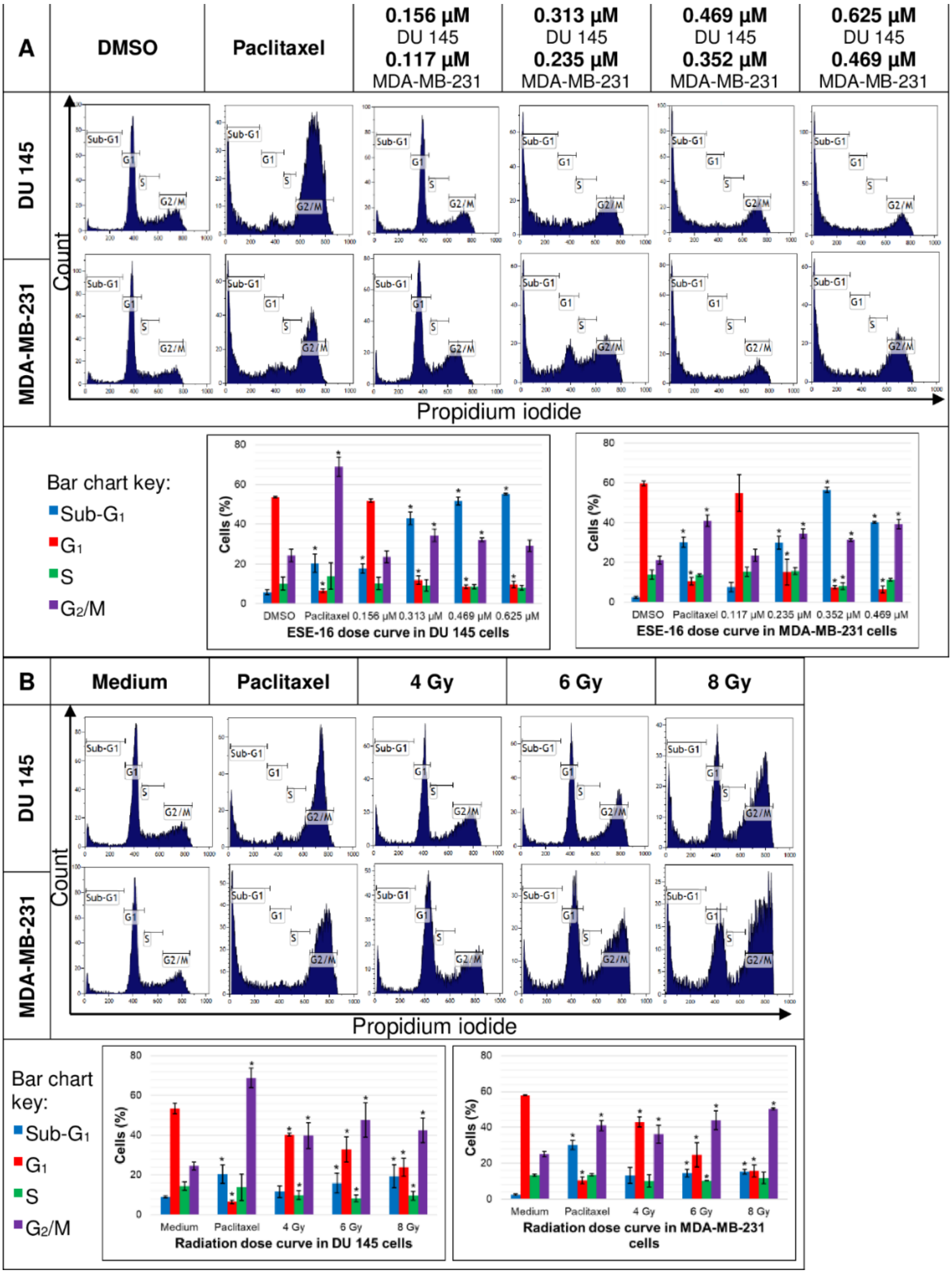

Figure S1. Experimental set-up using cell cycle analysis. Flow cytometric histograms of cell cycle progression in DU 145- and MDA-MB-231 cells exposed to (A) the ESE-16 concentration range and (B) the radiation dose range. Cell count was plotted against PI (FL3). DMSO and cells propagated in medium only served as vehicle- and negative controls respectively. ESE-16 concentrations between ¼ GI50 (0.156 µM in DU 145 cells and 0.117 µM in MDA-MB-231 cells) and ½ GI50 (0.313 µM in DU 145 cells and 0.235 µM in MDA-MB-231 cells) and a radiation dose of 4 Gy revealed notably higher sub-G1- and G2/M populations. Bar charts represent the means of three biological repeats with SD indicated by T-bars. Statistical significance (P value < 0.05) indicated by * when compared to DMSO or cells propagated in medium only.

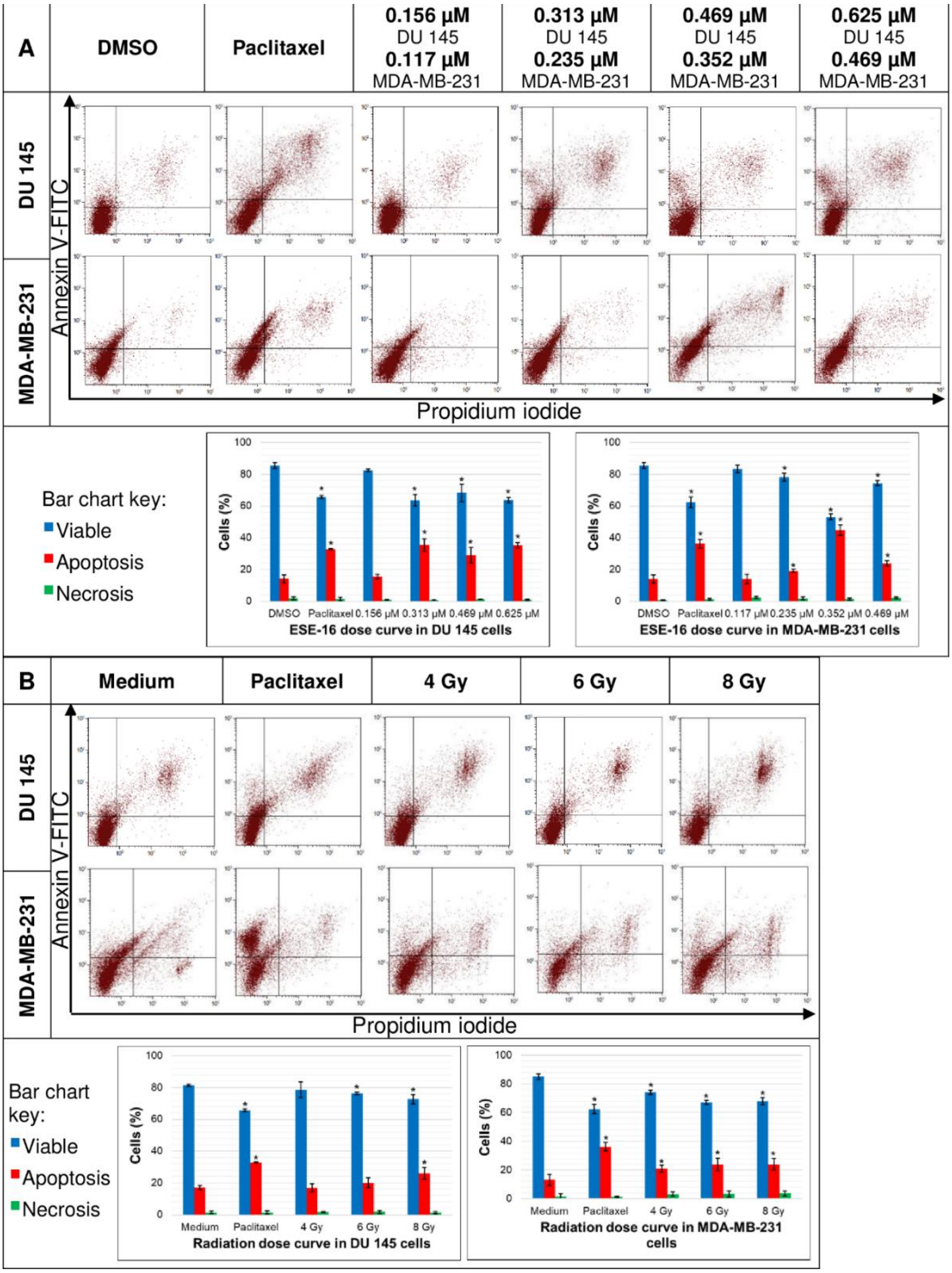

Figure S2. Experimental set-up using Annexin V-FITC apoptosis detection. Flow cytometric dot plots with Annexin V-FITC (FL1) plotted against PI (FL3) of DU 145- and MDA-MB-231 cells exposed to (A) the ESE-16 concentration range and (B) the radiation dose range. Paclitaxel served as a positive control for apoptosis. ESE-16 concentrations between ¼ GI50 (0.156 µM in DU 145 cells and 0.117 µM in MDA-MB-231 cells) and ½ GI50 (0.313 µM in DU 145 cells and 0.235 µM in MDA-MB-231 cells) and a radiation dose of 4 Gy revealed notable apoptosis induction with a concurrent reduction in cell viability. Bar charts represent the means of three biological repeats with SD indicated by T-bars. Statistical significance (P value < 0.05) indicated by * when compared to DMSO or cells propagated in medium only.
